# Treatment of cytokine storm syndrome with IL‐1 receptor antagonist anakinra in a patient with ARDS caused by COVID‐19 infection: A case report

**DOI:** 10.1002/ccr3.3307

**Published:** 2020-09-15

**Authors:** Leonard Kaps, Christian Labenz, Daniel Grimm, Andreas Schwarting, Peter R. Galle, Oliver Schreiner

**Affiliations:** ^1^ Department of Internal Medicine I University Medical Center of the Johannes Gutenberg‐University Mainz Germany; ^2^ Institute of Translational Immunology University Medical Center of the Johannes Gutenberg‐University Mainz Germany

## Abstract

The biological anakinra appears promising to halt cytokine storm syndrome seen in severe courses of COVID‐19. However, immunosuppression with anakinra may facilitate sepsis, necessitating continuous screening for bacterial superinfections.

## Graphical abstract

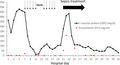

## 1 INTRODUCTION

Cytokine storm syndrome (CSS) represents a severe complication in COVID‐19. Anakinra, an Il‐1 receptor antagonist, may orchestrate the excessive inflammation. We report the use of anakinra (100 mg/d) in COVID‐19. Under therapy, clinical stabilization occurred. After discontinuation of the biological, bacterial superinfection with sepsis occurred.

On December 31, 2019, the number of hospitalized patients with severe pneumonia suddenly increased in the Chinese city of Wuhan (Hubei Province). Soon after the first cases, on January 7, 2019, Chinese health authorities reported that the outbreak was caused by a novel coronavirus, named coronavirus SARS‐CoV‐2.^1^ Epidemiologic data indicated that person‐to‐person transmission of SARS‐CoV‐2 was occurring, and the resulting disease was named coronavirus Disease 2019 (COVID‐19).^1^ Despite rigid containment and mitigation strategies by the Chinese government, infection rates increased and led to a pandemic. The currently reported overall case fatality rate is approximately 2%, but dramatically increasing up to 86% in patients with need for mechanical ventilation.^2^, ^3^

The infection may result in pneumonia going hand in hand with specific symptoms like fever, cough, dyspnea, and fatigue and may progress from mild‐to‐severe illness, while severe courses are not solely restricted to elderly or prediseased people.^1^ Yet, there is no specific antiviral therapy or vaccine available for COVID‐19. However, preliminary data of remdesivir, which was originally developed to treat HIV infections, showed promising results.^4^

In some cases, COVID‐19 may lead to severe immunologic complications such as macrophage activation syndrome (secondary hemophagocytic lymphohistiocytosis), resulting in cytokine storm syndrome (CSS) and acute respiratory distress syndrome (ARDS).^5^ Pathophysiologically, the immune system recognizes the viral antigens via antigen‐presenting cells and presents them to the natural killer and CD8‐positive cytotoxic T cells through major tissue histocompatibility (MHC) antigens. This may result in an excessive activation of both innate and adaptive immunity, which may lead to a release of a large amounts of pro‐inflammatory cytokines and chemokines.^5^ One hallmark of severe COVID‐19 courses is lymphopenia with low counts for T lymphocytes, including CD4, CD8 subtypes, and regulatory T cells (Tregs).^5^ In contrast, total leukocyte and neutrophil counts and neutrophil/lymphocyte ratio (NLR) including monocytes and macrophages are increased especially in severe cases.^5^ Although the underlying biological mechanism is not yet fully understood, the relative high abundancy of macrophages may explain elevated levels of pro‐inflammatory cytokines such as interleukin IL‐6, IL‐1, tumor necrosis factor TNF‐α, and IL‐8.^5^ Recently, in severe cases of CSS in patients with COVID‐19, tocilizumab, a humanized IgG1 monoclonal antibody to the IL‐6 receptor, has been used as a potential therapy to mitigate the excessive inflammatory response and showed some benefit in patients with severe COVID‐19 pneumonia.^6^, ^7^ Anakinra, a human interleukin‐1 receptor antagonist protein, represents an alternative to block severe systemic inflammation and has been suggested for the treatment of CSS in severely ill patients.^8^ This current case report describes the use of anakinra in a COVID‐19 patient with ARDS and CSS.

## 2 CASE REPORT

On March 20, 2020, a 53‐year‐old female Caucasian was admitted to the emergency room of the University Medical Center Mainz, Germany. Upon admission, she reported a history of 4 days of cough with persistent fever (>38.5°C) and progressing dyspnea. Since symptoms were in line with respiratory infections caused by COVID‐19, quarantine measures were put in place, following the internal guidelines for airborne disease. After initial assessment by a healthcare provider in the emergency room, she was transferred to intensive care unit (ICU).

**Figure 1:**
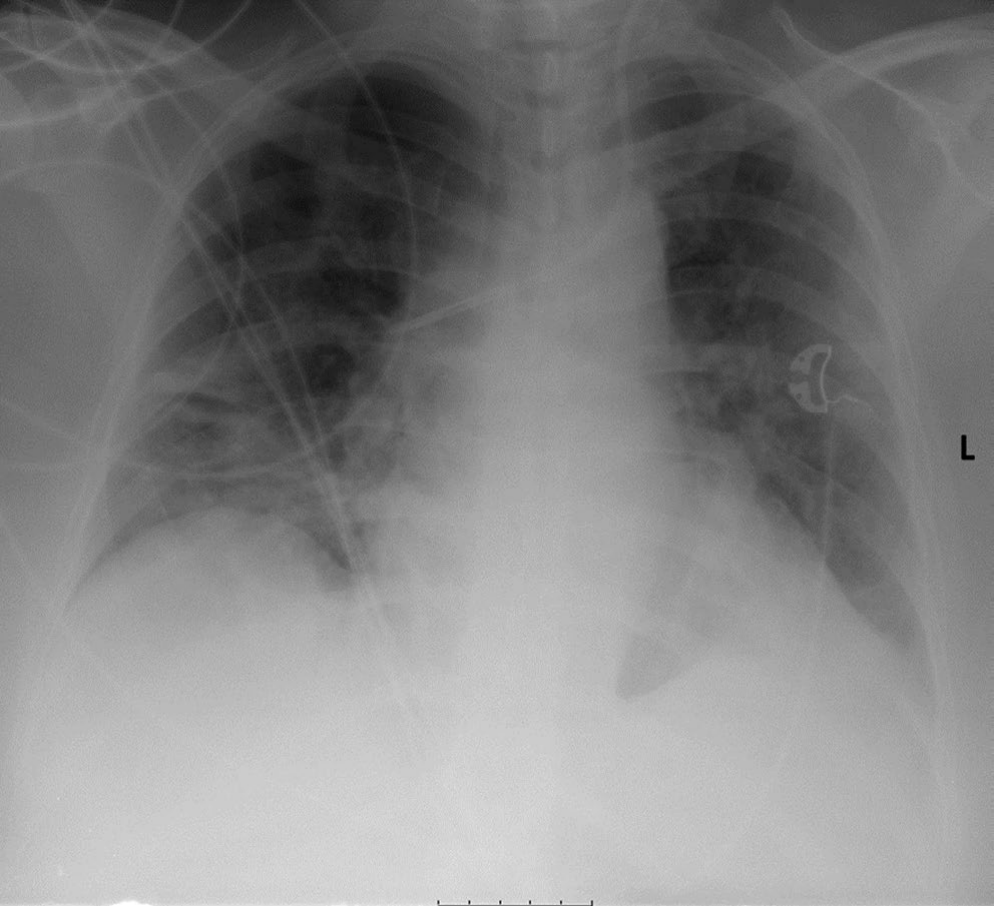
Anteroposterior chest X‐ray on admission: X‐ray showed diffuse bilateral infiltrates

The patient presented with fever of 38.9°C, blood pressure 127/73 mm Hg, pulse 90/min, a respiratory rate of 22/min, and a peripheral capillary oxygenation 94% while on 10 L/min oxygen therapy by face mask. Chest radiograph showed severe bilateral pulmonary infiltrates in the upper and lower lobes (Figure 1).

She weighed 90 kg at a height of 164 cm with a body mass index (BMI) of 33.5 (moderately obese) and reported having an alcohol abuse disorder, but discontinued drinking 6 months ago. Medical history included an asymptomatic, nonprogressing meningeal tumor, noninsulin‐dependent diabetes mellitus type 2, and arterial hypertension. Furthermore, hepatic steatosis and a psychiatric borderline syndrome were documented. Prescribed medication was metformin and antihypertensive medication.

Laboratory tests at hospital admission exhibited elevated inflammatory markers including CRP (242 mg/dL) in the absence of leukocytosis or procalcitonin (PCT). Additional parameters were mild lymphopenia of 15.6% and elevated levels of D‐dimer (0.66 mg/dL).^1^ Deep vein thrombosis (DVT) and pulmonary embolism (PE) were excluded by ultrasound or CT scan, respectively. Laboratory results are summarized in Table 1. Virologic testing, from nasopharyngeal swab and tracheal secrete, detected SARS‐CoV‐2 RNA by rapid nucleic acid amplification test (NAAT). Multiplex PCR assays for influenza, RSV (human respiratory syncytial virus), human parainfluenza, human metapneumovirus (HMPV), adenovirus, herpes simplex virus, cytomegalovirus (CMV), and microbiological testing of blood samples were negative.

**Table 1:** Laboratory results at different time points during treatment in the intensive care unit

	Admission	Anakinra day 1	Anakinra day 5	Anakinra PAUSE	Anakinra day 6	Anakinra day 9	Anakinra STOP	Onset of sepsis
Hospital day	1	7	11	12	14	17	18	22
C‐reactive protein (CRP) (mg/dL)	242	530	31	13	71	64	79	411
Leukocytes (/nL)	4.64	5.65	7.01	7.97	9.02	9.74	8	22.5
Procalcitonin (PCT) (ng/mL)	0.16	550	1.4	0.68	0.24		0.09	208
Ferritin (ng/mL)			3995		2051	968		
Ferritin (ng/mL)			3995		2051	968		
Triglyceride (mg/dL)		373					167	
Hemoglobin (g/dL)	11.6	10.4	8.6	8.2	7.6	8.3	8.4	8.6
Thrombocytes (/nL)	147	364	437	461	408	665	668	177
Creatine kinase (CK) (U/L)	154	668	420	305	253	233	113	104
Troponin I (pg/mL)	4	550	172	3.6	66	1.5	2.2	857

Hydroxychloroquine, as compassionate use, was initiated on day one and stopped on day 5 because of drug‐induced long QT syndrome.

Within 12 hours of admission, hypoxia worsened indicated by a spO2 of 64 mm Hg in the arterial blood when still breathing oxygen enriched air. At GCS (Glasgow Coma Scale) score of ≤8, the patient required mechanical ventilation. Despite mechanical ventilation was optimized, arterial oxygen saturation remained low (Horowitz index <150 mm Hg) so that intermittent prone positioning was initiated.

**Figure 2:**
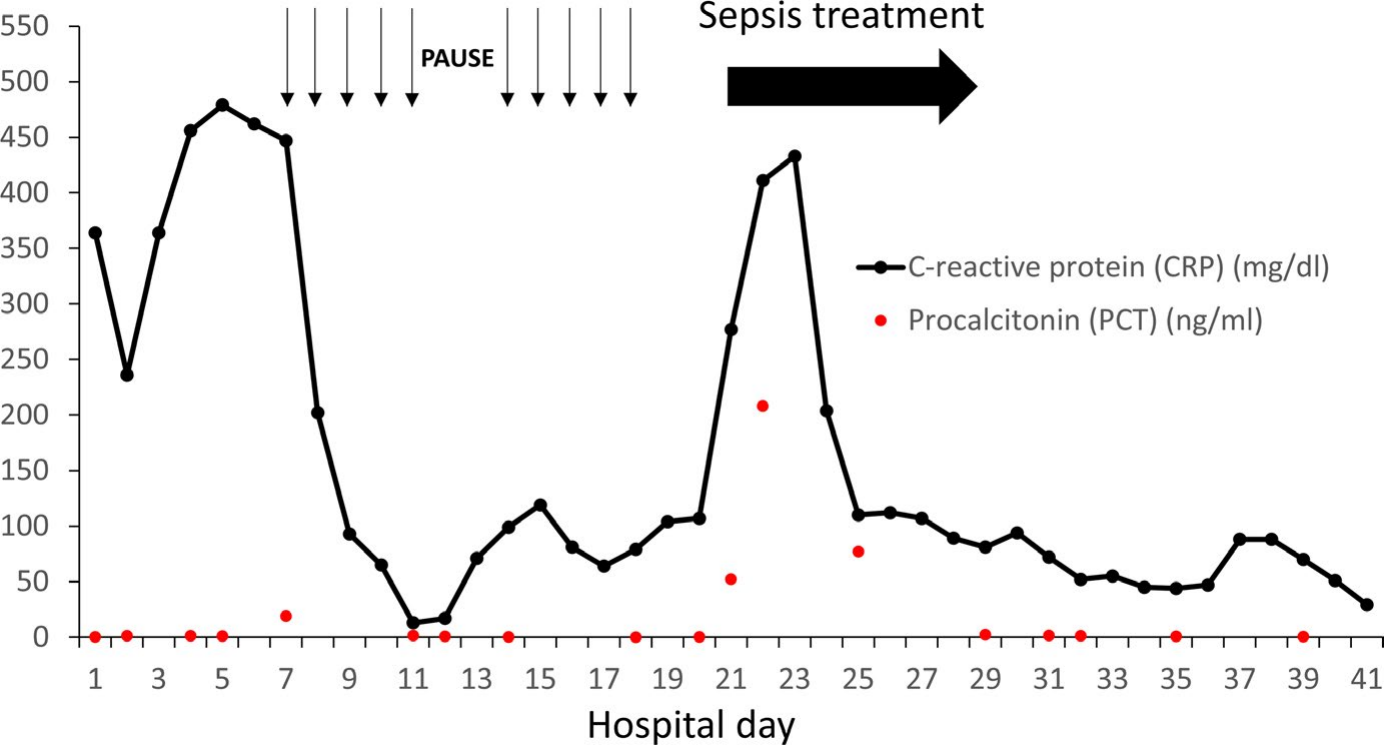
Course of the inflammatory markers CRP and PCT since admission of the patient. Arrows indicate administration of anakinra (100 mg s.c.). Antibiotic prophylaxis was administered from days 4 to 14

On day 7 following admission, she developed therapy refractory high fever (40°C), and high CRP (242 mg/dL) without elevated levels of PCT and negative microbiology, so CSS and a reactive HLH were suspected (calculation of HScore suggested 40%‐54% probability for HLH) despite missing hemophagocytosis in bone marrow aspiration. Serologic testing for autoimmune diseases (antinuclear antibodies (ANAs), extractable nuclear antigens (ENAs), antineutrophil cytoplasmic antibodies (ANCAs), anti‐dsDNA antibodies, and complement) was negative. The recombinant IL‐1 receptor antagonist anakinra (100 mg s.c.) was administered from days 7 to 11 together with antibiotic prophylaxis until day 14. Fever and inflammatory markers decreased substantially the next day, while the respiratory situation improved the following days (Figure 2). On hospital day 12, anakinra was discontinued for 48 hours on a trial basis resulting in relapsing inflammatory markers and fever on day 14. Consequently, the biological was reinstalled until day 18 with once again clinical improvement. In total, the patient received nine doses of anakinra over 12 days (Figure 2).

**Figure 3:**
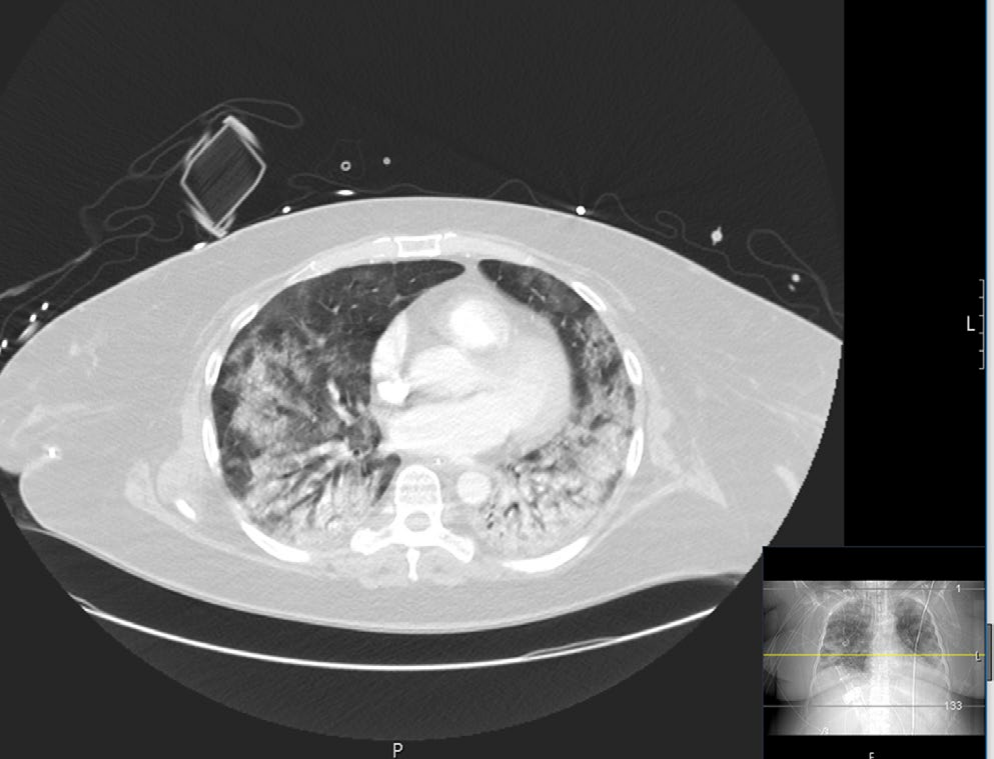
CT lung scan on day 20: CT scan shows severe bilateral infiltrates with lower and dorsal segments heavier affected

Bronchoscopy and chest CT were performed on hospital day 19. The CT scan presented with persistent bilateral pulmonary infiltrates with a predominant affection of the dorsal segments (Figure 3). During the same day, the clinical condition of the patient worsened again with signs of sepsis including hypotension (mean arterial pressure (MAP) <60 mm Hg) and impaired oxygenation. Empiric antibiotic therapy, consisting of meropenem and ciprofloxacin, was initiated. Microbiologic cultures reported positive for klebsiella pneumoniae in peripheral blood (time‐to‐positivity ~5 hours) and in bronchoalveolar lavage (BAL). Additionally, proteus mirabilis was detected in BAL. On day 22, CRP and PCT values were massively elevated with 411 mg/dL and 208 ng/mL, respectively. Moreover, continuous hemodiafiltration had to be initiated due to acute renal failure. Antibiotic therapy was adapted to ciprofloxacin according to the antibiogram, and inflammatory markers normalized together with improvement of circulatory and respiratory parameters. Renal replacement therapy was deescalated to intermittent hemodialysis and was finally stopped due to improvement of renal function. SARS‐CoV‐2 RNA in nasopharyngeal swab and BAL could not be detected anymore in consecutive samples. Due to long‐term mechanical ventilation, tracheotomy was performed on hospital day 27. Currently, the patient is breathing spontaneously without mechanical support and was discharged for postacute care at a rehabilitation center. The patient will be invited for follow‐up visits at our pulmonary disease outpatient clinic.

## 3 DISCUSSION

In this current report, we present a case of a patient with a severe course of COVID‐19 with accompanying CSS and HLH. Efforts to better understand the pathophysiology of COVID‐19, especially in severe cases, are currently ongoing. Excessive cytokine release syndrome has been observed in case series and is discussed as one of the underlying key components in severe COIVD‐19.^8^ This phenomenon is well known in different variations in sepsis, rheumatologic diseases, and HLH. The most vigorous form is named CSS, characterized by an overproduction of immune cells and their activating compounds like cytokines. Interestingly, this complication occurred also in previous pandemic flu infections (SARS‐CoV, MERS‐CoV) and is often associated with a surge of activated immune cells into the lungs, consequently leading to ARDS.^9^

The use of immunomodulators to attenuate the inflammatory cascade seems to be an appealing therapeutic approach. On the other the hand, an impairment of the immune system may delay viral clearance and bears the risk of bacterial superinfections.^10^ Biologicals including the immunomodulators anakinra or tocilizumab which selectively block the IL‐1‐receptor or IL‐6 receptor, respectively, appear to be promising for a more selective approach. The latter appeared to be beneficial to control excessive cytokine release in COVID‐19 as demonstrated in recent smaller single‐center studies.^6^, ^7^ In a recent retrospective cohort study, treatment with anakinra was demonstrated to be safe and was associated with lower mortality in patients with COVID‐19 and ARDS (90% survival in the anakinra treated group vs 56% in the standard treatment group, *P* = .009).^11^ However, it has to be acknowledged that this study was published after the therapy decision in our currently presented case was made. Our rationale of using anakinra over tocilizumab was its well‐studied and favorable safety profile and its shorter half‐life (with administration on a daily base) that offers adequate management especially if patients are at high risk of bacterial superinfection as in COVID‐19. As the condition of the patient deteriorated, all available therapeutic options for CSS were considered in an interdisciplinary consultation with our specialists of the rheumatology department. The application of steroids and azithromycin was considered but finally rejected as no data on steroids were available at this time, and azithromycin may cause abnormal changes in the electrical activity of the heart. Indeed, recent data suggested no benefit of the use of azithromycin in COVID‐19.^12^

After administration of anakinra, the clinical condition of the patient together with laboratory markers improved. However, the immunologic control of the inflammation seemed to come at the cost of bacterial superinfection with sepsis occurring after administration of anakinra. Data based on the German Biologics JIA Registry (BIKER) indicated that the use of anakinra contains a significant risk for infections in rheumatic disease.^13^ Additionally, bacterial superinfections are frequently seen in hospitalized patients undergoing ventilation for ARDS in viral diseases. A systematic review revealed that 25% of H1N1 patients during the pandemic in 2009 had a bacterial superinfection, which was associated with worse prognosis.^14^ Whether anakinra promoted the bacterial superinfection in this case remains hypothetical, and further investigation in clinical trials is urgently needed. Another limitation of this report is that the clinical improvement of the patient cannot be ascribed to the use of anakinra for sure as this report is based on a single observation only.

In conclusion, this report highlights the use of anakinra to mitigate the excessive inflammatory response in ARDS caused by COVID‐19. Additionally, this case demonstrates that patients treated with anakinra should be cautiously screened for bacterial infections and an antibiotic prophylaxis should be critically evaluated. Ongoing clinical trails should take a close look at the efficacy of anakinra on cost of potentially life‐threatening bacterial infections.^15^

## CONFLICT OF INTEREST

The authors disclose no potential financial or nonfinancial conflict of interests.

## AUTHOR CONTRIBUTIONS

LK, CL and OS wrote the manuscript. LK, CL, DG, AS, and OS were the treating physicians and edited the manuscript. PRG edited the manuscript and provided materials and scientific advice.

## ETHICAL APPROVAL

In line with the ethical guidelines at the institute and the federal law in Rhineland‐Palatinate (Germany), ethical approval was not sought for the present study because of the retrospective design of the study (case report).

## CONSENT STATEMENT

Written informed consent was obtained from the patient before publication.
